# Family Adjustment to Hereditary Cancer Syndromes: A Systematic Review

**DOI:** 10.3390/ijerph19031603

**Published:** 2022-01-30

**Authors:** Pedro Gomes, Giada Pietrabissa, Eunice R. Silva, João Silva, Paula Mena Matos, Maria Emília Costa, Vanessa Bertuzzi, Eliana Silva, Maria Carolina Neves, Célia M. D. Sales

**Affiliations:** 1 Cancer Genetics Group, Research Centre of IPO Porto (CI-IPOP)/RISE@CI-IPOP (Health Research Network), Portuguese Oncology Institute of Porto (IPO Porto)/Porto Comprehensive Cancer Centre (Porto.CCC), 4200-072 Porto, Portugal; esilva@ipoporto.min-saude.pt (E.R.S.); joao.pinho.silva@ipoporto.min-saude.pt (J.S.); marianeves@fpce.up.pt (M.C.N.); 2 Centre for Psychology at University of Porto (CPUP), Faculty of Psychology and Education Sciences at University of Porto (FPCEUP), 4200-135 Porto, Portugal; pmmatos@fpce.up.pt (P.M.M.); ecosta@fpce.up.pt (M.E.C.); elianasilva@fpce.up.pt (E.S.); celiasales@fpce.up.pt (C.M.D.S.); 3 Department of Psychology, Catholic University of the Sacred Heart, 20123 Milan, Italy; Giada.Pietrabissa@unicatt.it (G.P.); vanessa.bertuzzi@unicatt.it (V.B.); 4 Psychology Research Laboratory, IRCCS Istituto Auxologico Italiano, 20122 Milan, Italy; 5 Psychology Service, Portuguese Oncology Institute of Porto, 4200-072 Porto, Portugal; 6 Medical Genetics Service, Portuguese Oncology Institute of Porto, 4200-072 Porto, Portugal

**Keywords:** hereditary cancer syndromes, genetic testing, family adjustment, cancer risk management, decision-making, genetic counseling

## Abstract

Hereditary cancer syndromes are inherited pathogenic genetic variants that significantly increase the risk of developing cancer. When individuals become aware of their increased probability of having cancer, the whole family is affected by this new reality and needs to adjust. However, adjustment to hereditary cancer syndromes has been mainly studied at an individual level, and research about familial adjustment remains dispersed and disorganized. To overcome this gap, this review aims to understand how families adjust to genetic testing and risk management, and to what extent the family’s adjustment influences the psychological response and risk management behaviors of mutation carriers. We conducted searches on the PubMed/Med Line, PsycInfo, SCOPUS, and Google Scholar databases and used the Mixed Methods Appraisal Tool (MMAT-v2018) to assess the methodological quality of each selected study. Thirty studies met the inclusion criteria. Most results highlighted the interdependent nature of adjustment of pathogenic variant carriers and their families. The way carriers adjust to the syndrome is highly dependent on family functioning and related to how family members react to the new genetic information, particularly partners and siblings. Couples who share their worries and communicate openly about cancer risk present a better long-term adjustment than couples who use protective buffering (not talking about it to avoid disturbing the partner) or emotional distancing. Parents need help dealing with disclosing genetic information to their children. These findings reinforce the importance of adopting a family-centered approach in the context of genetic counseling and the necessity of involving family members in research.

## 1. Introduction

Nearly five to ten percent of all cancers are hereditary and caused by genetic pathogenic variants that significantly increase the lifetime risk of cancer when compared to the general population [1]. To identify whether an individual carries a pathogenic variant that increases the risk of cancer, geneticists may prescribe genetic tests for cancer susceptibility to individuals from hereditary cancer families [2,3]. When a healthy individual is identified as a pre-symptomatic pathogenic variant carrier, cancer risk may be reduced through enhanced surveillance procedures (e.g., regular colonoscopies), pharmacy chemoprevention, and/or prophylactic surgeries (e.g., mastectomies) [4,5]. Additionally, family planning solutions, such as medically assisted reproduction, may be offered to prevent future children from carrying the pathogenic variant [6]. However, although preventing cancer onset is crucial for pathogenic variant carriers, knowing about future cancer risk, deciding on the personal prevention plan, and adapting to risk-reduction measures pose significant psychological challenges [7,8,9,10,11]. In fact, deciding if, how, and when to undergo the invasive medical procedures usually reserved for these situations [4,12] may not only elicit distress in the pathogenic variant carriers [13] but also in their family members [14].

Therefore, the aftermath of a positive genetic testing result commonly represents a critical event for the whole family [15]. Not only as it has the potential to impact the health of family members who may also be carrying the pathogenic variant, but it may also affect family members’ relationships and a couple’s reproductive decisions [16,17]. In addition, genetic testing uptake is usually carried out in a cascading way [18], which often means that the new pathogenic variant carriers vicariously experience the outcome of their carrier relatives’ decisions [14]. In this sense, dealing with the uncertainty of possible future losses without abdicating from important life cycle goals (e.g., having children) requires pathogenic variant carriers and their family members to develop great flexibility in an inter-related adjustment process [19,20]. In other words, to understand the psychological adjustment to inherited cancer risk, one must conceive it as a systemic phenomenon [19,20,21].

Previous systematic reviews have already summarized the existing knowledge concerning the psychological adjustment of the applicant to genetic testing and counseling [22,23,24,25,26,27,28] and on the effectiveness of interventions to help pathogenic variant carrier health-related decision-making processes [29]. However, studies on familial adjustment in the face of inherited genetic syndromes remain dispersed and disaggregated. To our knowledge, to date, only one review [30] focused on familial adjustment to hereditary cancer syndromes. Additionally, this contribution only explored the experiences of male partners of hereditary breast and ovarian cancer syndrome carriers.

Intending to address this gap, the present systematic review organizes current knowledge on the topic of familial adjustment to hereditary cancer syndromes in a rigorous and replicable way. Our objective is to review findings from existing research investigating the psychological adjustment of the families of pre-symptomatic pathogenic variant carriers to an increased susceptibility to hereditary cancer. Specifically, we focused on the period following a positive genetic test, including the long-term adjustment to personalized prevention programs to understand: (1) how do families adjust to a positive genetic test result of a pre-symptomatic member and his/her risk management behaviors? (2) to what extent does the family influence the psychological adjustment and risk management behaviors of the pre-symptomatic pathogenic variant carrier?

## 2. Method

This systematic review was registered with PROSPERO (ID CRD42020142200). Data extraction, critical appraisal, and qualitative synthesis followed established systematic review and qualitative synthesis methods [31] and the Preferred Reporting Items for Systematic Reviews and Meta-Analyses (PRISMA) statement [32].

### 2.1. Search Strategy

We conducted searches in the following databases: PubMed/MEDLINE, Scopus, PsycInfo, and Google Scholar between 28 June to 2 July 2019, which were updated between the 13 and 17 January 2020, and again between 23 June and 2 July 2021 and complemented with hand-searching. The search strategies combined key terms and Medical Search Headings (MESH) terms for the concepts of inherited cancer risk, unaffected pathogenic variant carrier, emotional distress, family psychosocial adjustment and risk management behavior, and personalized preventive programs, following the PICO elements [33]. Boolean and truncation operators were used to combine search terms more systematically and to list documents containing variations on search terms, respectively [34]. We modified the search syntax as appropriate for each database.

### 2.2. Inclusion and Exclusion Criteria

We only included articles that: (1) were original studies; (2) described the psychosocial adjustment of the family system of pre-symptomatic pathogenic variant carriers to inherited cancer risk test and/or personalized preventive programs; (3) investigated at least one measure of psychological adjustment (e.g., emotional distress, quality of life, anxiety, depression, anger) to genetic testing results or risk management behaviors of carrier’s family members; (4) investigated family barriers and facilitators of decision making and adherence to personalized preventive programs/risk management behaviors. Studies targeting families of either mixed populations (affected/unaffected) or currently unaffected individuals previously diagnosed with cancer were included. We did not restrict publications based on the study design, ethnicity, or year of publication. We opted only to include studies on pre-symptomatic pathogenic variant carriers to better capture the experience of being and living with a pathogenic variant carrier, which may be different from that of being and living with a hereditary cancer patient. The methodological choice of not restricting studies by design methods aimed to aggregate all existing knowledge about the psychological adjustment of families to inherited cancer risk. Articles were excluded if: (1) pre-symptomatic pathogenic variants carriers were under the age of 18 years; (2) considered families were made up only of individuals not yet tested for inherited cancer risk; (3) considered families of affected (cancer patients) were solely pathogenic variant carriers; (4) only biomedical outcomes were considered; (5) only the psychosocial adjustment of pre-symptomatic pathogenic variant carriers was investigated, but not one of their family members.

### 2.3. Study Selection

Following the search and exclusion of duplicates, two reviewers (authors GP and VB) independently screened the eligibility of the articles first by title and abstract, and the full text according to the inclusion criteria. Authors CS and PMM resolved disagreements. Authors PG and CS conducted a separate search by independently screening reference lists of previous systematic reviews [22,23,24,26,28,30,35], one doctoral thesis [36], and searching articles in relevant journals (e.g., American Journal of Human Genetics, European Journal of Human Genetics, Journal of Genetic Counselling, Journal of Community Genetics, Health Psychology, Psycho-Oncology). Search updates were conducted by author PG in 2020 and by authors PG and MN in 2021. Following Smith et al. [37], the review team included at least one person with methodological expertise in conducting systematic reviews (PG, GP, VB, CS, PMM) and at least two experts on the topic under review (CS, ES, EC, JS).

**Figure 1:**
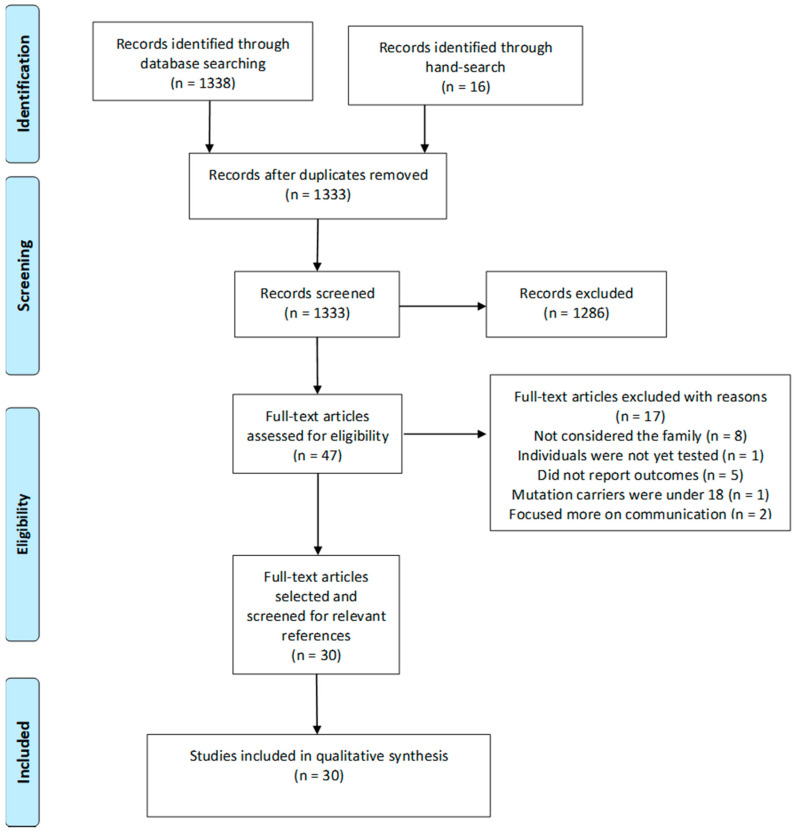
PRISMA Flow-chart.

Searches of electronic databases identified 1338 reports, and searches by hand identified 16 articles. From the 1354 total records found, 21 were duplicates, and 1286 records were excluded based on information from the title and abstract. The remaining 47 articles were evaluated for inclusion by reviewing their full text and resulted in the exclusion of 17 records for the following reasons: (1) considered only the psychosocial adjustment of unaffected pathogenic variant carriers, but not of their families (*n* = 8) [38,39,40,41,42,43,44,45]; (2) considered the psychological adjustment of families of individuals not yet tested for inherited cancer risk; (*n* = 1) [46]; (3) did not report quantitative/qualitative outcomes (e.g., opinion, theoretical, perspective articles, etc.) (*n* = 5) [47,48,49,50,51]; (4) pre-symptomatic pathogenic variant carriers were under the age of 18 (*n* = 1) [52]; (5) study focuses more on the carrier’s type of communication than on the psychological impact of the genetic test results on their family/system (*n* = 2) [53,54]. During the search update, three new articles were included [55,56,57]. References of the remaining 30 articles were further screened for relevant records, but none was found. The flowchart presented in Figure 1 provides step-by-step details of the study selection process.

### 2.4. Assessment of Risk of Bias

The mixed-methods appraisal tool (MMAT-v2018) [58] was used to assess the methodological quality of each selected study. The MMAT-v2018 includes a total of 25 criteria and two screening questions rated on a scale of yes, no, and cannot tell. The tool results in a methodological rating of 0 (0% lowest quality) to 5 (100%, highest quality) quality criteria met by each study [58]. The assessment was conducted independently by authors VB and GP, and any disagreements were resolved by a third author (PG, PMM, or CS). The ratings of each selected study are presented in Supplementary material 1 (Table S1).

### 2.5. Data Extraction and Synthesis

Authors GP and VB independently extracted the following data from included studies: first author and year of publication; country; aim of the study; study design; study setting; study outcome; follow-up points, sample size, age of applicants; gender of the applicants; clinical status of the family member; the role of the member in the family; the family role of the pathogenic variant carriers; type of pathogenic variant/cancer; psychological/behavioral outcome(s) and measure; results; retention; barriers and facilitators. The results of selected studies were summarized to provide a qualitative synthesis of the impact that genetic risk test positive results and risk management decisions have on the family psychosocial adjustment, and how the family influences the psychological response and risk management behaviors of both the pre-symptomatic pathogenic variant carrier and family members themselves.

## 3. Results

### 3.1. Description of the Included Studies: The MMAT-v2018 Checklist

The distribution of MMAT-v2018 scores was generally high across study designs, with 22 out of 30 (73.3%) studies meeting 100% of quality criteria and eight records having 80% of quality criteria met (see Supplementary Table S1). All qualitative studies (*n* = 5) [14,56,59,60,61] obtained the highest possible score, which was also achieved in 3 out of 7 (42.9%) quantitative non-randomized studies [62,63,64] and 13 out of 17 (76.5%) records adopting a quantitative descriptive method [55,65,66,67,68,69,70,71,72,73,74,75,76]. One mixed-method study of the two included in this review (50%) also met 100% of the quality criteria [77]. No quantitative randomized controlled trial was included.

### 3.2. Overview of Characteristics of Included Articles

We identified 30 studies meeting inclusion criteria. Most of them (*n* = 17) were conducted in the USA, while five studies were carried out in the Netherlands [62,65,66,71,78], two in Canada [67,79], two in Australia [66,68], one in the United Kingdom [69], one in Portugal [56], one in France [57], and another one in Japan [63]. Twenty-one records focused only on hereditary breast and ovarian cancer syndrome (HBOC); three focused on hereditary non-polyposis colorectal cancer syndrome (HNPCC) [59,63,80]; three studies comprised a sample of both HBOC and HNPCC pathogenic variant carriers [65,66,81]; one study included HNPCC, HBOC, and hereditary diffuse gastric cancer syndrome (HDGC) pathogenic variant carriers [56]; and one study regarded Familial Adenomatous Polyposis [71]. The sample size varied from a minimum of 17 participants (cumulative between pathogenic variant carriers and their family members) [79] to a maximum of 272 pathogenic variant carriers, only [65,66]. Carriers were all adults in the age range of 20–50. Most studies investigated the experience of genetic testing in couple and sibling relationships. When referring to psychological adjustment, studies considered a variety of constructs, such as general and cancer-related distress, cancer worry, risk perception, depression, anxiety, and somatization. Most papers presented pre- post-testing studies conducted with pathogenic variant carriers, with post-testing typically measured six months after testing results. However, one study measured long-term adjustment of up to 24 months after receiving genetic testing results [72]. Five papers employed a qualitative research method conducted on pathogenic variant carriers and their family members [14,56,59,60,61]. A summary of the characteristics of selected studies can be found in Table 1, while a more complete description of selected records can be consulted in Supplementary Table S2.

### 3.3. The Family System Facing Genetic Testing Results

Overall, family members experienced anguish, worry, and guilt after genetic testing results throughout the selected studies. More specifically, partners of pathogenic variant carriers reported psychological distress, anxiety [68,71,76,77], and worries about their spouse developing cancer and dying, as well as concerns about their children carrying the pathogenic variant [67]. Adult daughters of unaffected BRCA1/2 (a type of HBOC) carriers showed clinical levels of cancer-related distress and worries both regarding their mothers and other relatives [77].

Moreover, pathogenic variant carriers disclosed feeling guilty for their children and their carrier siblings [63,66], while non-carrier HNPCC applicants declared feeling guilty towards relatives with hereditary cancer [63]. Also, both carriers and non-carriers showed worries about relatives who opted not to pursue genetic testing [63,66]. Untested women potentially at risk of HBOC presented less psychological distress and decisional conflicts when compared to their relatives who pursued genetic testing [55]. However, they were also less knowledgeable about potential risk factors and gene inheritance, besides reporting a greater perception of disease severity and controllability [55].

#### 3.3.1. Positive and Negative Relational Changes after Genetic Testing

Applicants for HBOC and HNPCC reported feeling closer to their partners and siblings, while communication, support, and appreciation also improved for the other relatives. At the same time, applicants reported familial conflicts and difficult situations within the family due to genetic testing [66]. A study comparing HBOC carriers and non-carriers found that perceptions of family expressiveness (the extent that family members are encouraged to act openly and express their feelings) decreased significantly more for carriers between the first genetic counseling session and 9 months after results [84]. In contrast, perceptions of family cohesion and family conflict did not differ significantly between pathogenic variant carriers and non-carriers [84]. Interestingly, one study observed that in families with HBOC and HNPCC, an increase in the levels of family conflict after genetic testing predicted lower depression scores at 12 months [70].

Three reviewed studies indicate that pre-existing family functioning characteristics are predictors of the psychological adjustment of applicants [65,66,70]. HNPCC applicants who perceived lower family cohesion at pre-test, as well as a decrease in family cohesion six months after receiving the results, had higher depression scores one year later [70]. Also, applicants who perceived their nuclear families as disengaged or enmeshed at pre-test presented higher levels of hereditary cancer distress six months after test results [65]. They also experienced more adverse consequences in their relationships with partners and children when compared to applicants who felt their families moderately cohesive and adaptive [66].

The presence of the pathogenic variant in the extended family was also a predictor of the psychological adjustment of newly discovered pathogenic variant carriers. A study with 179 first and second-degree relatives of HNPCC pathogenic variant carriers found that a higher number of carriers (both in the immediate and extended family) predicted more depressive symptomatology in pathogenic variant carriers six months after the test result [80]. Cancer-related worries also tended to rise with the increase in the number of HNPCC carriers in the extended family, and cancer-related distress and worry were higher when the proportion of carriers in the immediate family was low [71]. Discrepancies in cancer worry (when applicants had significantly different results in cancer worry at pre-test compared to the family norm) also predicted higher depression scores 12 months after HNPCC results [70].

#### 3.3.2. Siblings’ Adjustment

Four studies included in this review focused specifically on the adjustment dynamics within sibships [73,74,75,78]. Smith et al. [75] assessed the psychological distress before and after genetic testing for BRCA1/2 in 212 sibships tested simultaneously. Distress was higher for both siblings when one sibling tested positive alone, and lower when results were positive for more than one sibling. Moreover, non-carrier siblings felt more distress when their siblings were carriers than when all siblings were non-carriers [75]. The importance of the pathogenic variant pattern among brothers and sisters was also discovered by Hamann et al. [74], who concluded that siblings who both tested positive for BRCA1/2 reported more friendly behavior towards each other than when only one sibling did. Also, Lodder et al. [78] found that non-carrier women who had a sister recently identified with the BRCA 1/2 pathogenic variant presented higher levels of depression at post-test than other non-carrier women. Furthermore, a study with 65 sisters of HBOC families found that non-carriers experienced similar levels of perceived risk, cancer worry, anxiety, and somatization as carriers [73].

#### 3.3.3. Couples’ Adjustment

Eleven studies focused specifically on the adjustment process to test results regarding couples [59,61,64,66,67,68,69,71,72,82,83]. Cross-over effects in the couple were observed. That is, emotional states in one member of the couple were found to affect the adjustment of the other. Partner’s distress was correlated with the level of distress reported by women at risk of developing breast/ovarian cancer [68] and individuals with familial adenomatous polyposis [71]. High levels of partner support reported by HBOC carriers before taking the test significantly predicted lower levels of pathogenic variant carriers’ distress up to two years after the test [72]. On the other hand, perceived partner anxiety predicted pathogenic variant carrier distress in the same time frame [72]. Importantly, effect sizes were stronger when pathogenic variant carriers perceived their partners as both unsupportive and anxious before testing, which predicted clinical distress levels up to two years after testing [72]. In another study focusing on HBOC couples, applicants reported less general and cancer-specific distress six months after test results when their partners had shared concerns and had provided effective support before genetic testing [82]. Moreover, low perceived partner support and the partner avoiding talking about worries to protect the partner (protective buffering) predicted higher general distress in the applicant [82]. However, some applicants actively avoid partner support. Oostrom et al. [66] found that some applicants react to a positive pathogenic variant test by trying to emotionally distance themselves from their partners to protect and prepare them for the possibility of developing cancer or dying for it.

Six studies reported on the impact of the pathogenic variant in the partner and the couple’s relationship [59,61,67,68,82,83]. Partners of HBOC pathogenic variant carriers experienced genetic testing as a stressful situation and considered this new medical condition exacerbated pre-existing couple relationship problems [61]. These partners indicated they would need more support but did not search for it because they were focused on helping their applicant spouses [61]. Some partners also mentioned discomfort about sharing worries with their spouses [61,82] because they did not want to overburden them. Therefore, the couple did not share their preoccupations, which often led applicants to think their partners were not sufficiently caring [61,67]. In this context, partners reported significant relationship strain [82], less couple intimacy, more frequent discussions about the future [83], and a sense of urgency regarding the pursuit of couples’ life goals [61]. In contrast, open communication about cancer risk within the couple was found to be related to less distress in partners of women at risk for developing breast/ovarian cancer [68]. Some BRCA 1/2 partners reported becoming more involved in their spouses’ families, talking more about cancer risk with the pathogenic variant carrier [83], and feeling closer to their spouses after genetic testing [67]. Also, some partners of HNPCC pathogenic variant carriers and partners of not-yet-tested HNPCC individuals did not perceive their partners’ medical condition as personally relevant, even if their children were also at risk of carrying the pathogenic variant [59].

Findings from two studies suggest that approaching cancer risk as a team may benefit the couple’s adjustment [61,69]. Study [69] found a significant positive association between dyadic consensus (the extent to which couples agree on important matters for the relationship) and levels of perceived support in couples dealing with HBOC risk. Higher scores for support and team approach were also significantly associated with the couple’s satisfaction69. Another study with HBOC families found that dealing with cancer risk as a team facilitates psychological adjustment, along with more active participation from partners in decision-making processes, greater satisfaction with their role as a supporter, and optimism [61]. However, the positive effect of dealing with cancer risk as a team was not confirmed by Shapira et al. [64], who did not find a significant association between dyadic coping and psychological adaptation in women identified with the BRCA 1/2 pathogenic variant.

#### 3.3.4. The Family System Facing Risk-Reduction Decision Making and Long-Term Managing of Cancer Risk

Six studies [14,56,59,60,62,64] focused specifically on family participation in risk reduction decision-making processes and long-term adjustment to life with hereditary cancer risk. A qualitative study with a heterogeneous hereditary cancer risk population found that family members generally communicated during involvement in genetic counseling and adopted open communication within the nuclear family and first-degree relatives but reported more difficulty informing extended family relatives due to emotional distance [56]. Moreover, BRCA1/2 pathogenic variant carriers interviewed by Puski et al. [14] reported that having witnessed family members’ experience of being diagnosed and treated for HBOC represented a stimulus to be pro-active and to opt for risk-reducing surgery. They considered it helpful to gain information about the surgical process, healing time, and side effects from family members who had already undergone a mastectomy or an oophorectomy [14]. The participants reported that while non-carrier relatives gave support by validating decisions, pathogenic variant carriers provided camaraderie and instilled hope and reassurance by sharing their experiences [14]. However, two participants reported adverse effects concerning support given by relatives who either tried to persuade them to undergo the surgical procedure when they did not want to or expressed disapproval when they intended to do the surgery [14].

Two studies found that risk management decisions had a significant impact on the process of adaptation to life with increased cancer risk, both for the pathogenic variant carriers and for their partners [62,64]. Study [64] discovered that women who underwent a risk-reducing mastectomy and their partners reported significantly higher levels of psychological adaptation to BRCA1/2 than women who did not. Study [62] explored the long-term psychological impact of either regular breast cancer surveillance or prophylactic surgery and found that open communication within the nuclear family (partner and children) and with the family of origin (parents and siblings) four to nine years after genetic testing results were significantly associated with less breast-cancer-related distress in BRCA 1/2 pathogenic variant carriers [62]. Open communication with family members also significantly mediated the positive effect of social support on general and breast cancer-specific distress. In other words, women who felt supported by their families were more likely to talk openly about hereditary cancer with their close relatives (e.g., partner, children, parents, and siblings), promoting their psychological adjustment [62].

Regarding the risk management of unaffected non-carriers, those that belong to BRCA1/2 families report overscreening behaviors, even though these were not recommended [57]. These overscreening behaviors correlated to a higher feeling of self-vulnerability and higher comparative pessimism (e.g., tendency to think that negative events will more likely happen to oneself than to others), but not to higher anxiety levels [57].

#### 3.3.5. Parents and Children Facing Hereditary Cancer Risk

Three studies reported on the experience of being a parent in a family identified with hereditary cancer syndromes [60,76,77]. Two of three studies focused on the impact that poor communication might have on children of parents who are pathogenic variant carriers [60,76]. A qualitative study [60] involving five families with HBOC showed that parents found it difficult to talk with their children about cancer risk or downplayed the importance of the information and postponed disclosing their status. Consequently, children ended up learning about the familial cancer risk in an informal manner, without really understanding its meaning and consequences [60]. Children’s inaccurate cancer risk perception and poor health literacy were also found by Patenaude et al. [77]. Some adult daughters of BRCA1/2 pathogenic variant carriers were unaware that they could be at a higher risk for cancer because their mothers were pathogenic variant carriers. Also, they did not know that if a woman carries the pathogenic variant, her sister has a 50% probability of being a carrier, and they were unaware of the importance of undergoing genetic testing to prevent cancer onset [77]. The parental influence on children’s future health behavior was evident in HBOC families. It was observed that daughters made the same choice their pathogenic variant carriers’ mothers did regarding both genetic testing and risk management measures [60].

Difficulties in discussing cancer risk with their children also affected pathogenic variant carriers significantly [76]. In a study by Mays et al. [76] poor parent-child communication before genetic testing predicted greater distress in mothers one month after receiving their HBOC test results [76]. Interdependent effects of parenting dynamics were also reported in women at risk of HBOC and their partners. More specifically, high levels of pre-test individual uncertainty for one parent regarding how to communicate test results to children significantly predicted increased distress in the other member of the couple up to one month after test results [76].

One study reported that some adult descendants of HBOC carriers viewed the likelihood of being a pathogenic variant carrier as an intense experience, eliciting internal conflicts and emotional pain [77]. Although most daughters in this study did not perceive themselves as less healthy because their mothers were carriers, others revealed deep fears and concerns regarding the possibility of carrying the pathogenic variant. These included worrying about their psychological health, pessimistic and paranoid thoughts, as well as the fear of having to change life plans, regarding childbearing in particular. Moreover, some daughters were worried about transmitting the pathogenic variant to their children or not living long enough to see them grow up. Many expressed the intention of undergoing genetic testing as soon as possible, but others refused to be tested; the majority expressed ambivalence [77].

**Figure 2:**
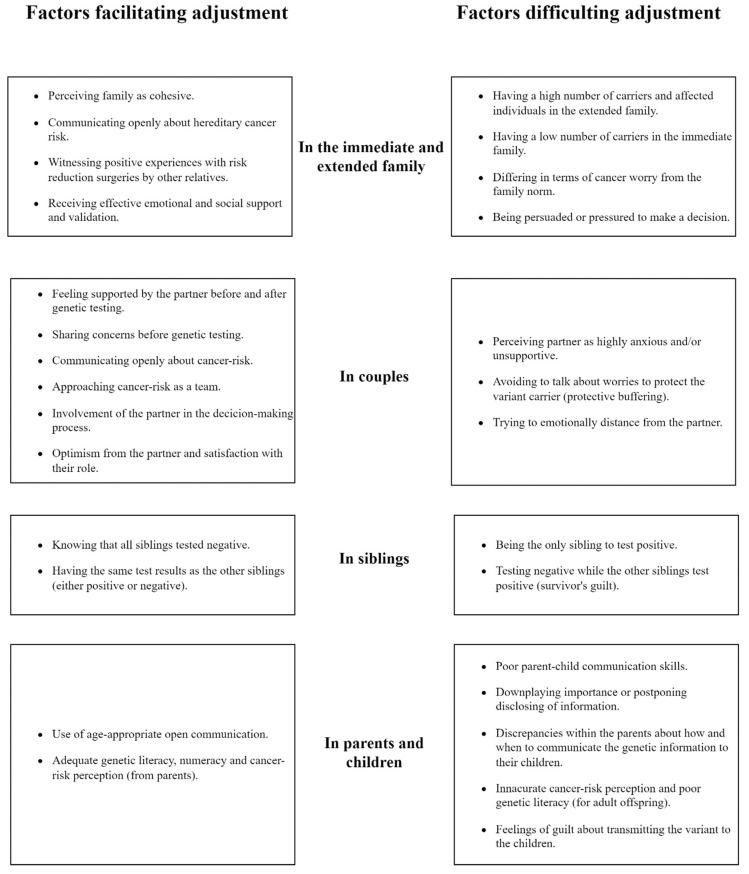
Summary of the factors affecting family adjustment to hereditary cancer syndromes.

A summary of all the factors we found that affect adjustment to hereditary cancer syndromes within the family system can be found in Figure 2.

## 4. Discussion

This systematic review intended to synthesize how families with pathogenic variant carriers unaffected by cancer adjust to genetic testing results and life-saving risk management programs offered in the context of cancer genetic counseling. Taken together, the findings reveal a complex panorama where the systemic nature of family adjustment is evident. The diagnosis of a hereditary cancer syndrome in an unaffected individual works as a collective stressor, affecting not only the applicant but also their family. As posited by Daly [20], the hereditary cancer syndrome goes from generation to generation, impacting each family member and the entire family because “a positive genetic test would challenge the family identity” (p. 549). As such, family members who are carriers may experience depression, anxiety, and distress due to the fear of developing cancer and dying from it, as well as concerns about transmitting the pathogenic variant to their children. Moreover, non-carrier relatives may feel guilty towards family members affected by a pathogenic variant or cancer disease, thus experiencing a particular kind of “survivor guilt” [85] (p. 321). Survivor guilt is a distressing experience, where individuals blame themselves for being the ones to survive a life-threatening situation or disease where others perished got injured, or critically ill [85]. It is common in trauma [85] and cancer survivors [86], and it can be a threat to families’ well-being and systemic functioning as it may lead to relational distancing. Nevertheless, a diagnosis of hereditary cancer syndrome can also bring positive changes. Findings show that being a pathogenic variant carrier seems to bring a particular sense of connection and togetherness with other carriers in the family, possibly due to sharing similar distressing experiences [87]. This translates to relational changes in several sub-systems. Some family members might experience greater closeness, improved communication patterns, and supportive dynamics, while others face conflicts and difficult situations [66].

Reviewed studies also highlighted that pathogenic variant carriers may adjust differently to positive genetic testing results, depending on the history and presence of the syndrome in the nuclear and extended family [80]. These findings are in line with the Family Systems Illness model (FSI; [19]), which is increasingly applied in genomic disorders as a theoretical framework for understanding family challenges. According to this model, successful or dysfunctional family adaptation depends on the psychosocial demands of the disorder and the family functioning and the resources used to cope with this stressor by the family members. This family adaptation seems to be particularly important at the level of close relationships, i.e., between siblings, partners, and children. Selected studies provided evidence of better individual adjustment to the result of genetic testing when siblings have the same results, even if they test positive. Furthermore, both carrier and non-carrier siblings appear to experience similar levels of perceived risk, cancer worry, anxiety, and somatization [73], revealing the adjustment processes to be interdependent. Regarding the partners of pathogenic variant carriers, although some of them did not consider the medical condition of their partners as personally relevant [59], others viewed genetic testing as a stressful event that seemed to exacerbate previous problems in the romantic relationship [62]. Some partners described their need for support. However, their role as caregiver and support provider for their partner led them not to share their feelings and concerns [62,82]. In addition, the couple faces a central distressing difficulty in sharing genetic risk information with their children and in dealing with their reactions. For the children, the likelihood of also being a pathogenic variant carrier appeared as an intense experience they needed to adjust to [77]. To this end, children stressed the importance of more accurate information about genetic risk and what it entails so they could make informed decisions about their health and future life plans. This review also recognized the parents’ difficulties and children’s need for information that led Werner -Lin et al. [88] to develop a set of recommendations for healthcare professionals to facilitate parents’ decisions and disclosure processes for their school-aged children, adolescents, and young adults, according to their developmental abilities.

Family functioning and adjustment to a positive genetic test result and risk management behaviors of a pathogenic variant carrier also influenced the psychological adjustment and decision-making of new carriers. Some family dynamics (e.g., lower levels of family cohesion, disengagement, enmeshment) before and after genetic testing were associated with individuals’ depression [70], higher levels of cancer distress, and worse relationships with partners and children [65] after receiving test results. The reviewed studies also provided strong evidence for the impact of the partner’s adjustment on the pathogenic variant carrier’s long-term fine-tuning to the hereditary cancer syndromes. For example, communication about the syndrome was hindered when carriers felt their partners were unsupportive, not involved, or not attuned to their suffering, and thus preferred to cope with the stressful situation alone [59,66]. In contrast, open communication and approaching cancer as a team was associated with better adjustment [61,62,68]. This is in line with a previous review in which the interaction between couple members influences each other’s distress [30] and in studies with couples facing cancer, where it is suggested that couples may act as systems, not just as individuals [89].

Although the literature is scarce, our review suggests that pathogenic variant carriers followed the example of their family members, deciding in terms of genetic testing and risk-management options. Family members might positively influence individual decisions and risk management behavior of those carrying a pathogenic variant, functioning as role models, and through sharing first-hand experiences or as interlocutors thus support the decision-making process. Testimonials and active encouragement from family members might also provide valuable information to help potential applicants decide whether to undergo genetic testing and other preventive measures [14,90]. Nevertheless, relatives might also function as a source of biased information or contribute to additional distress when they insist on trying to persuade pathogenic variant carriers to make certain decisions, challenging their self-determination [14].

Support received from family members was recognized by both non-carriers and carriers as important in dealing with the challenges posed by their condition. Emotional support in families with hereditary cancer, in which multiple members simultaneously provide and receive support from relatives, was characterized as a communal coping process [73,91], as previously identified in individuals with other chronic conditions [92]. The positive effect of family support on distress seems to be explained by open communication between family members [62]. However, communication may be hindered by protective buffering processes. Pathogenic variant carriers may refrain from sharing their concerns for fear of aggravating the distress of their close relatives or may emotionally slip away from their partners to help them better prepare for the eventuality of their death from cancer [66]. Indeed, this dimension has already been recognized in previous studies, leading to the development of interventions specifically aimed to improve family communication about hereditary cancer and genetic testing [93].

### 4.1. Limitations of the Current Research

Most of the selected studies focused on the psychological adjustment of families with inherited hereditary breast and ovarian cancer risk and HNPCC risk. Only one study explored the emotional response and role of the family in the decision-making process of positive mutation carriers of other syndromes (Familial Adenomatous Polyposis). This may reflect a gap in the literature on the topic, but also that our search entailed a syntax that was not broad enough to encompass other hereditary cancer syndromes. Other medical terms may have been used in studies relating to other hereditary cancer syndromes. Also, not restricting studies by study design helped aggregate existing knowledge but prevented a meta-analytical approach that could yield a more precise estimate of the strength of the findings.

### 4.2. Research Gaps and Recommendations for Future Studies and Clinical Practice

Our review identified four major research gaps. First, there is scant knowledge about the psychological adaptation of children growing up in families with a known hereditary cancer syndrome. This calls for further research that explores how hereditary cancer affects the psychological development and health behaviors of children and adolescents, and how it should be managed to minimize negative impacts. A second major gap concerns the lack of research focused on the long-term adjustment phases of risk management. Little is known about how best to involve family members at critical turning points in risk-reduction decision-making and their adjustment to the consequences of those decisions over time. In this process, it is important to consider the limits and possible contraindications of family involvement. Such knowledge is critical to empowering the family to use its resources for mutual support.

A third gap concerns the lack of cancer genetic health literacy interventions for both applicants and their relatives on the following topics: the multiple psychological impacts of hereditary cancer risk on applicants, their biological relatives, and partners; the expected impact on relationships and how to overcome it; the importance of family self-supportive dynamics (e.g., open communication), and the common difficulties associated with it (e.g., protective buffering, survivor guilt). Health literacy targeting close relatives from whom the carrier expects support, such as partners, might be more important in families without a clinical history of familial cancer, given the possible devaluation of the syndrome and the carrier’s distress. Fourth and lastly, there is a lack of translational research towards family-centered genetic cancer risk care. The reviewed role of the family in the individual adjustment of pathogenic variant carriers calls for a debate about balancing the right to self-determination and individual privacy (e.g., the right not to involve the family in decision-making processes) with family-centered interventions. Hereditary cancer syndrome is a clinical condition in which several members of the same family are at different stages of diagnosis/risk management through interdependent adjustment processes and communal coping. Thus, interventions that target the family system and not just its members individually should be considered, to better address the family’s psychosocial and clinical needs. The few existing interventions, for example, Multifamily Discussion Groups (MFDG) [21], have shown promising preliminary evidence regarding improving family relationships and psychological well-being.

Research should further focus on the multiple interrelated adjustment processes [15] to better capture the systemic nature of family adaptation. To this end, we provide four methodological recommendations. First, to include family members as informants. Most studies have used the applicant as the only data source, but it is important to examine the lived experience of family members, especially children and young people. Second, to examine the process of interdependent adjustment of family members (e.g., couples, siblings, parents-children sub-systems) through dyadic or triadic study designs. Third, use of longitudinal designs to examine family dynamics and adjustment trajectories. Studies should consider the major family events expected in the course of hereditary cancer syndromes: including the pathogenic variant carrier or a family member developing cancer; the pathogenic variant carrier or a family member undergoing prophylactic surgery; the pathogenic variant carrier reaching a critical age (e.g., the age at which the index case was diagnosed with cancer); the pathogenic variant carrier becoming a parent; a child of the pathogenic variant carrier reaching a critical age (e.g., the age at which they must decide whether or not to be tested); or the death of a family member diagnosed with cancer. Fourth, the active involvement of pathogenic variant carriers and their families in research using participatory approaches would ensure that research focuses on topics relevant to patients and close to their daily experiences. Moreover, this may contribute not only to informing health professionals and improving care practices but also to increasing people’s participation and adherence to treatment.

## 5. Conclusions

To our knowledge, our review is the first to describe family member adjustments to genetic testing and risk management, and its influence on the psychological response and risk management decisions and behaviors of pathogenic variant carriers. Our findings provide valuable evidence regarding the interdependent adjustment processes of family members to the increased risk of developing cancer. Three family members emerged as particularly important for adult pathogenic variant carriers’ coping with the inherited genetic pathogenic variant: siblings, romantic partners, and children. In fact, hereditary cancer syndrome is a familial health condition, and a positive genetic testing result in a person might have implications for their close and extended relatives. Their relationships change, and pre-existing family functioning may affect not only the adjustment of the newly diagnosed pathogenic variant carrier over time but may also subsequently affect the adherence to genetic testing and the health behavior of other relatives. It is therefore important to educate and support healthcare professionals in identifying key strengths and weaknesses in individual and family adjustments for the provision of family-centered care.

## Figures and Tables

**Table 1:** Characteristics of the included studies (summarized).

Author, Year	Design	GT	Sample Size (*n*)	Gender|MALE-*n*(%):FEMALE-*n*(%)	Main Outcomes
Ashida et al., 2009 [70]	Longitudinal cohort study	HBOC	178	75(42.1%):103(57.9%)	Lower perceived family cohesion associated with higher depression scores; Increase in family conflict associated with lower depression scores in families with higher levels of cancer worry.
Bartle-Haring et al., 2003 [81]	Longitudinal observational (pilot) study	HBOC or HNPCC	50:MC = 25; FM = 25	MC = 5(20%):19(76%); FM = 9(36%):14(56%)	Higher levels of differentiation of self are associated with less distress in mutation carriers and family members.
den Heijer et al., 2011 [62]	Longitudinal observational	HBOC	222	F = 100%	Open communication within families is associated with less breast cancer-specific distress and plays a mediating role between social support and distress.
Di Prospero et al., 2001 [79]	Exploratory study	HBOC	24	2(8.3%):22(91.7%)	Most participants felt a little or moderately worried about cancer risk, and nine subjects considered they would benefit from a support group.
Douma et al., 2011 [71]	Cross-sectional	FAP	FM = 129	63(49%):65(51%)	30% of partners reported moderate to severe levels of distress; Partners ‘distres significantly associated with carriers’.
Eliezer et al., 2014 [80]	Longitudinal observational	HNPCC	179 (26 families)	75(42%):104(58%)	A higher proportion of carriers in the family predicted a higher probability of participants presenting clinical levels of depression
Hamann et al., 2008 [74]	Experimental	HBOC	98 (49 dyads: 16 positive, 13 negative, 20 mixed results)	23(23.5%):75(76.5%)	Dyads with mixed results (one positive, one negative) reported less friendly support behavior and a higher increase in anger than dyads with positive or negative results.
Katapodi et al., 2011 [55]	Descriptive, Cross-sectional	HBOC	372 (MC = 200; FM = 172)	F = 100%	Probands showed higher risk perception and more distress than their relatives; Relatives showed a higher perception of severity and controllability.
Koehly et al., 2008 [73]	Cross-sectional	HBOC	65 (31 families)	F = 100%	Significant within family correlation of perceived risk, cancer worry, anxiety, and somatization irrespective of mutation status.
Lodder et al., 2001 [78]	Longitudinal observational	HBOC	154 (MC = 78 FM = 56)	F = 100%	Higher anxiety levels were found in 20% of carriers and 35% of partners. Levels of anxiety best predicted by pre-test level of anxiety.
Manne et al., 2004 [82]	Longitudinal observational	HBOC	464 (MC = 212; FM = 252)	MC_F = 100%; FM = 117(99.2%):1(0.8%)	Less partner support and more protective buffering from partner before the test predicted more distress from carriers 6 months after results; Partners who felt understood by applicants at baseline reported less distress 6 months after.
Mauer et al., 2015 [83]	Cross-sectional	HBOC	FM = 25		Participants reported negative changes in intimacy levels, attraction, and communication with their partners and more frequent discussions about the future.
Mays et al., 2014 [76]	Prospective study	HBOC	109 dyads	FM_Mothers: F = 100%; FM_Partners: M = 100%	Decisional conflicts before genetic testing from one member of the couple predicted higher distress in the other member of the dyad one month after the test.
McInerney-Leo et al., 2005 [84]	Prospective study	HBOC	262	Total = 92(35%):170(65%)	Perceptions of family cohesion increased both when participants underwent testing and when they did not; Conflict decreased from baseline for those who underwent testing.
Mendes & Sousa, 2012 [56]	Exploratory, qualitative study	HNPCC or HBOC or HDGC	50 (9 families)	F = 58%	Cancer related events within the family impact how carriers assess their risk; Families consider genetic counseling an emotionally taxing process.
Metcalfe et al., 2002 [67]	Cross-sectional	HBOC	FM = 59	M = 100%	Twenty percent of partners considered their carrier spouse received inadequate support. Most partners felt that the syndrome brought them closer to their spouse
Milhabet et al., 2013 [57]	Cross-sectional	HBOC	FM = 77	F = 100%	Overscreening behaviors by non-carriers were associated with feelings of self-vulnerability and pessimism related to cancer risk
Mireskandari et al., 2006 [61]	Exploratory study	HBOC	FM = 15	M = 100%	Better adjustment and coping for partners of women with HBOC were associated with dealing with the stressor as a team, involvement in the decision-making, satisfaction with supportive role, and optimism.
Mireskandari et al., 2007 [68]	Single-assessment study design	HBOC	190 (MC = 95; FM = 95	95(50%):95(50%)	Clinical levels of distress were reported by 10% of partners of women at high risk of HBOC; Open communication within the couple associated with less partner distress.
Murakami et al., 2004 [63]	Prospective qualitative study	HNPCC	47 (MC = 31; FM = 16)	Total = 20(47.6%):22(52.4%)	Some carriers reported feelings of guilt either towards their children or family members affected by cancer.
Norris et al., 2009 [60]	Descriptive qualitative study	HBOC	17 (5 families)	6(35.3%):11(64.7%)	Families often need more professional support than what they are getting with genetic counseling. Parents are unsure about how to share genetic information with their offspring.
Patenaude et al., 2013 [77]	Observational mixed-methods study	HBOC	MC = 40	F = 100%	Daughters presented worries about their own risk and their mothers’, and 32% percent of participants showed clinical levels of cancer-risk distress.
Peterson et al., 2003 [59]	Retrospective qualitative study	HNPCC	39 (5 families)	15(38.5%):24(61.5%)	Spouses of carriers considered the news about the mutation as less personally relevant even when they had children at risk. Members of families with the most uptake of genetic testing worried for others that opted not to be tested
Puski et al., 2018 [14]	Qualitative descriptive	HBOC	20	F = 100%	Most often, family members are involved in the decision-making process by providing emotional and social support; Some family members may put too much pressure on carriers to make a decision, causing them discomfort.
Shapira et al., 2017 [64]	Observational	HBOC	229 (MC = 168; FM = 61)	NR	Partner’s perception of risk similar to carriers’. Dyadic coping scores not related to carriers’ or partners’ adaptation scores
Smith et al., 1999 [75]	Longitudinal observational	HBOC	212	87(41%):125(59%)	Non-carrier men whose siblings tested positive reported more distress than when siblings tested negative; Carrier women whose siblings either tested negative or had not yet been tested presented greater psychological distress.
Van Oostrom et al., 2007a [65]	Prospective study	HNPCC and HBOC	MC = 271	32(12%):239(88%)	Participants perceiving family functioning as maladaptive reported more hereditary cancer-related distress than participants who perceived their family as adaptive.
Van Oostrom et al., 2007b [66]	Prospective study	HNPCC and HBOC	MC = 272	32(12%):239(88%)	Perceiving their family as enmeshed-chaotic or disengaged and feeling less free to talk about cancer-risk related issues predicted relationship problems with their family.
Watts et al., 2011 [69]	Observational	HBOC	188 (MC = 94; FM = 94)	F = 100% (FM_M = 100%)	Higher perceived support associated with greater dyadic consensus and satisfaction; Dyadic cohesion and satisfaction were associated with the use of a team approach when dealing with stressors
Wylie et al., 2003 [72]	Longitudinal observational	HBOC	203	M = 100%	Higher anxiety from the partner predicts higher distress for the tested person, while higher support from the partner predicts lower distress for the applicant.

Legend: NR = GT = Genetic test; HNPCC = Hereditary nonpolyposis colorectal cancer; HBOC = Hereditary breast and ovarian cancer syndrome; FM = family member; MC = pathogenic variant carrier.

## Data Availability

Not applicable.

## Supplementary Materials

The following supporting information can be downloaded at: www.mdpi.com/article/10.3390/ijerph19031603/s1. The supplemental data includes two tables: Supplementary Table S1—Evaluation of quality of selected records according to the mixed-methods appraisal tool checklist (MMAT-v2018) and Supplementary Table S2—Detailed characteristics of the included studies.
