# Supplementary material for: Family Adjustment to Hereditary Cancer Syndromes: A Systematic Review
Source: Int J Environ Res Public Health. 2022 Jan 30;19(3):1603. doi: 10.3390/ijerph19031603 (PMC8834948; doi:10.3390/ijerph19031603)
Supplement: Supplementary file 1 [file ijerph-19-01603-s001.zip › Suplementary File 2 Table S2.pdf]

Supplementary file 2: **Characteristics of the included studies**

**Author, year**	**Country**	**Design**	**GT**	**Sample size (n)**	**Age (yrs) | mean (SD), range**	**Gender | MALE - n(%):FEMALE - n(%)**	**Main outcomes**
Ashida et al., 2009	USA	Longitudinal cohort study	HBOC	178	39.77(14.75), 18-83	75(42.1%):103(57.9%)	Lower perceived family cohesion associated with higher depression scores; Increase in family conflict associated with lower depression scores in families with higher levels of cancer worry.
Bartle-Haring et al., 2003	USA	Longitudinal observational (pilot) study	HBOC or HNPCC	50:MC=25; FM=25	MC=39.8; FM=40.8	MC=5(20%):19(76%); FM=9(36%):14(56%)	Higher levels of differentiation of self are associated with less distress in mutation carriers and family members.
den Heijer et al., 2011	NLD	Longitudinal observational	HBOC	222	47.1(8.3), 29–68	F=100%	Open communication within families is associated with less breast cancer specific distress and plays a mediating role between social support and distress.
Di Prospero et al., 2001	CAN	Exploratory study	HBOC	24	52.9, 31-77	2(8.3%):22(91.7%)	Most participants felt a little or moderately worried about cancer-risk and nine subjects considered they would benefit from a support group.

Douma et al., 2011	NLD	Cross-sectional	FAP	FM=129	FM=46.1 (11.5), 21-79	63 (49%):66 (51%)	30% of partners reported moderate to severe levels of distress; Partners ‘ distres significantly associated with carriers’.
Eliezer et al., 2014	USA	Longitudinal observational	HNPCC	179 (26 families)	39, 18-72	75(42%):104(58%)	A higher proportion of carriers in the family predicted a higher probability of participants presenting clinical levels of depression
Hamann et al., 2008	USA	Experimental	HBOC	98 (49 dyads:16 with positive results, 13 with negative results and 20 with mixed results)	TotalMean=47.72(12.99)	23(23.5%):75(76.5%)	Dyads with mixed results (one positive, one negative) reported less friendly support behaviour and a higher increase in anger than dyads with positive or negative results.
Katapodi et al., 2011	USA	Descriptive, Cross-sectional	HBOC	372 (MC= 200; FM=172)	51(11), 22–83	F=100%	Probands showed higher risk perception and more distress than their relatives; Relatives showed higher perception of severity and controllability.
Koehly et al., 2008	USA	Cross-sectional	HBOC	65 (31 families)	40.7(8.6)	F=100%	Significant within family correlation of perceived risk, cancer worry, anxiety and somatization irrespective of mutation status.

Lodder et al., 2001	NLD	Longitudinal observational	HBOC	154 (MC=78 FM=56)	MC=38.4; FM=38.7	F=100%	Higher levels of anxiety were found in 20% of carriers and 35% of partners. Levels of anxiety best predicted by pretest level of anxiety.
Manne et al., 2004	USA	Longitudinal observational	HBOC	464 (MC=212; FM=252)	MC=50(10.76), 27–75; FM=54, 29-79	MC_F=100%; FM=117(99.2%):1(0.8%)	Less partner support and more protective buffering from partner before test predicted more distress from carriers 6 months after results; Partners who felt understood by applicants at baseline reported less distress 6 months after.
Mauer et al., 2015	USA	Cross-sectional	HBOC	FM=25	FM=38.5, 24-55		Participants reported negative changes in intimacy levels, attraction and communication with their partners as well as more frequent discussions about the future.
Mays et al., 2014	USA	Prospective study	HBOC	109 dyads	FM_Mothers:45.9 (6.1); FM_partners:47.8 (6.9)	FM_Mothers:F=100%; FM_Partners:M=100%	Decisional conflicts before genetic testiing from one member of the couple predicted higher distress in the other member of the dyad one month after the test.
McInerney-Leo et al., 2005	USA	Prospective study	HBOC	262	TotalMean=40	Total=92(35%):170(65%)	Perceptions of family cohesion increased both when participants underwent testing

							and when they did not; Conflict decreased from baseline for those who underwent testing.
Mendes & Sousa, 2012	PT	Exploratory, qualitative study	HNPCC or HBOC or HDGC	50 (9 families)	NR	F=58%	Cancer related events within the family impacts how carriers assess their risk; Families consider genetic counseling an emotionally taxing process.
Metcalfe et al., 2002	CAN	Cross-sectional	HBOC	FM=59	FM=50.6, 28-73	M=100%	Twenty percent of partners considered their carrier spouse received inadequate support. Most partners felt that the syndrome brought them closer to their spouse
Milhabet et al., 2013	FR	Cross-sectional	HBOC	FM=77	FM=32.09(5.21)	F=100%	Overscreening behaviours by non-carriers were associated with feelings of self-vulnearability and pessimism related to cancer risk
Mireskandari et al., 2006	AUS	Exploratory study	HBOC	FM=15	41.4, 30–56	M=100%	Better adjustment and coping for partners of women with HBOC were associated with dealing with the stressor as a team, involvement in the decision-making, satisfaction with supportive role and optimism.

Mireskandari et al., 2007	AUS	Single-assessment study design	HBOC	190 (MC=95; FM=95	MC=45.4(10.2), 23-72; FM=42.9(9.4), 23-67	95(50%):95(50%)	Clinical levels of distress were reported by 10% of partners of women at high risk of HBOC; Open communication within the couple associated with less partner distress.
Murakami et al., 2004	JAP	Prospective qualitative study	HNPCC	47 (MC=31; FM=16)	TotalMean=47(10), 28-60	Total=20(47.6%):22(52.4%)	Some carriers reported feelings of guilt either towards their children or family members affected by cancer.
Norris et al., 2009	USA	Descriptive qualitative study	HBOC	17 (5 families)	NR	6(35.3%):11(64,7%)	Families often need more professional support than what they are getting with genetic counselling; Parents are unsure about how to share genetic information to their offspring.
Patenaude et al., 2013	USA	Observational	HBOC	MC=40	FM=21, 18-24	F=100%	Daughters presented worries about their own risk and their mothers'; Thirty two percent of participants showed clinical levels of cancer-risk distress.
Peterson et al., 2003	USA	Retrospective, cross-sectional,	HNPCC	39 (5 families)	TotalMean=49.2, 21-81	15(38.5%):24(61.5%)	Spouses of carriers considered the news about the mutation as less personally relevant even when they had children at risk. Members of families with the most uptake of genetic testing

		qualitative study					worried for others that opted to not be tested
Puski et al., 2018	USA	Qualitative descriptive	HBOC	20	20-50	F=100%	Most often, family members are involved in the decision-making process by providing emotional and social support; Some family members may put too much pressure on carriers to make a decision, causing them discomfort.
Shapira et al., 2017	USA	Observational	HBOC	229 (MC=168; FM=61)	TotalMean=39.7(10.1)	NR	Partner's perception of risk were similar to carriers. Dyadic coping scores was not related with carriers' or partners' adaptation scores
Smith et al., 1999	USA	Longitudinal observational	HBOC	212	TotalMean=46.32(16.72)	87(41%):125(59%)	Non-carrier men whose siblings tested positive reported more distress than when siblings tested negative; Carrier women whose siblings either tested negative or had not yet been tested presented greater psychological distress.
Van Oostrom et al., 2007a	NLD	Prospective study	HNPCC	MC=271	MC=43.25(12.7)	32(12%):239(88%)	Participants perceiving family functioning as maladaptive reported more hereditary cancer related distress than participants who perceived their family as adaptive.

Van Oostrom et al., 2007b	NLD	Prospective study	HNPCC	MC=272	MC=43.25(12.7)	32(12%):239(88%)	Perceiving their family as enmeshed-chaotic or disengaged and feeling less free to talk about cancer-risk related issues predicted relationship problems with their family.
Watts et al., 2011	UK	Observational	HBOC	188 (MC=94; FM=94)	MC=42.9(9.4), 23–67; FM=45.4(10.2), 23–72	F=100% (FM_M=100%)	Higher perceived support associated with greater dyadic consensus and satisfaction; Dyadic choesion and satisfaction were associated with the use of a team approach when dealing with stressors
Wylie et al., 2003	USA	Longitudinal observational	HBOC	203	TotalMean=45.27(13.67)	M=100%	Higher anxiety from the partner predicts higher distress for the tested person, while higher support from the partner predicts lower distress for the applicant.

***Supplementary file 1*: Characteristics of the included studies (continue)**

Author, year	MC Familiar role	FM	FM Gene Pathogenic variant Status	Follow-up points	Outcome:Measure	Quality
Ashida et al., 2009	NR	NR	Carrier-NonCarrier	T0: Baseline T1: 6 months after disclosure –	Depression:CES-D; Family relationships:FES; Cancer worry:ad hoc tool	5

				T2: 12 months after disclosure		
Bartle-Haring et al., 2003	Siblings, spouses, or son/daughter	Siblings, spouses, or parents	Carrier	T1: baseline – T2: at disclosure	Distress:IES; Depression and anxiety:HSCL; Differentiation of Self:DSI	4
den Heijer et al., 2011	Daughter, partner, mother, friend	NR	Carrier	T0: baseline – T1: 2 months	Distress:IES; Depression and anxiety:HADS; Social support:MSPSS	5
Di Prospero et al., 2001	Parents, partner	Partner, sons	NonCarrier	NR	Cancer risk perception; worry about cancer; attitudes toward surveillance and prevention options; satisfaction with clinical services; additional support - satisfaction of having taken genetic test:ad hoc tool	4
Douma et al., 2011	Partner	Partner	NonCarrier	NR	Cancer Worry: CWS, Intrusion: IES-Intrusion subscale, and Quality of Life: SF36 Health Survey	5
Eliezer et al., 2014	Partner, son, parent, cousin, uncle	Internal and external family	Carrier-NonCarrier	T0: baseline (genetic test) – T1: 6 months (test results delivered 1-2 months from baseline)	Distress:IES; Cancer worry and Depression:ad hoc tool	4
Hamann et al., 2008	Sibling	Sibling	Carrier-NonCarrier	NR	Circumplex measures:SAS-C, IMI-C; Anger and anxiety:STAI	5
Koehly et al., 2008	Sister	Sister	Carrier-NonCarrier	NR	Distress:BSI; Cancer worry:LCWS; Cancer risk perception:PRI; Social integration, reciprocity and shared support:ad hoc tool	5
Lodder et al., 2001	Partner	Partner	NonCarrier	T0: pre-test – T1: post-test	Depression and anxiety:HADS; Distress:IES	4
Manne et al., 2004	Partner	Partner	NonCarrier	T0: pre-test – T1: 6 months after disclosure	Partner Response to Cancer:PRCI; Distress:IES; Depression and anxiety:BSI-Anxiety and Depression subscales; Partner support; Relationship strain; Sharing of concerns:ad hoc tool	4
Mauer et al., 2015	Partner	Partner	NonCarrier	NR	Male attitudes towards partnership and sexuality:ad hoc tool	4

Mays et al., 2014	Mothers	Partner	Carrier-NonCarrier	T0: baseline – T1: 1 month after disclosure	Distress:BSI; Parent-Adolescent Communication:PAC; Decisional conflict:DCS.	5
McInerney-Leo et al., 2005	NR	NR	NonCarrier	T0: baseline – T1: 6 months – T2: 9 months	Family Relationship Index:FRI; Family Environment:FES	4
Mendes & Sousa, 2012	NR	NR	Carrier-NonCarrier	NR	Familial experience of genetic counseling and meaning, impacts and management of the genetic condition: ad hoc semi-structured interview; The personal relevance of the family interview: post-interview questionnaire, ad hoc tool	
Metcalfe et al., 2002	Partner	Partner	NonCarrier	NR	NR	5
Milhabet et al., 2013	NR	NR	NonCarrier	NR	Screening behaviors: ad hoc tool; State anxiety: STAI-YA; Feelings of self-vulnerability or self-risk perception: ad hoc tool; Comparative perception of risk or comparative optimism	
Mireskandari et al., 2006	Partner	Partner	NonCarrier	NR	NR	5
Mireskandari et al., 2007	Partner	Partner	NonCarrier	NR	Monitoring attentional style:MBSS; Distress, Depression and anxiety:DASS and IES; Information and support needs; Knowledge about breast/ovarian cancer; Individual and couple factors; Cancer–related event in the family:ad hoc tool.	5
Murakami et al., 2003	NR	NR	NonCarrier	T0: pre-test – T1: administration genetic test – T2: 1 month after test	Depression:SCID; Personality factors:EPQR; Feelings of guilt:ad hoc tool	5
Norris et al., 2009	Mothers and wives	Sons and husbands	NonCarrier	NR	Communication and decision-making strategies:ad hoc Semi-structured Interview	5
Katapodi et al., 2011	NR	NR	Relatives did not pursue testing	NR	Illness appraisals of HBOC: Breast/Ovarian cancer risk factor knowledge index; IPQ-R; ad hoc tool; Psychological distress: ad hoc tool; Family environment: Family problem solving and communication index; Family relationships inventory; Decisional Conflict: Decisional conflict scale	

Patenaude et al., 2013	Mothers	Daughters	Carrier-NonCarrier	NR	General distress: BSI-18; Cancer related distress: IES; Breast Cancer Genetic Counselling Knowledge: BCKQ	5
Peterson et al., 2003	NR	NR	Carrier-NonCarrier	NR	Personal perceptions and persuasion in motivating:ad hoc Semi-structured Interview	5
Puski et al., 2018	NR	Not specified	Carrier	NR	Decision-making:ad hoc Semi-structured Interview	5
Shapira et al., 2017	Partner	Partner	NonCarrier	NR	Psychological adaptation:PAS; Dyadic coping:DCI; Risk perception:ad hoc tool;	5
Smith et al., 1999	Sibling	Siblings	Carrier	T0: baseline, before genetic testing – T1: 1/2 weeks after receiving results	Distress:IES, STAI	5
Van Oostrom et al., 2007a	NR	NR	Carrier-NonCarrier	T0: baseline – T1: 1 week – T2: 6 months after disclosure	Distress:IES; Anxiety and Depression:HADS; Nuclear family functioning:FACES; Differentiation to parents:DFSC; Familial communication style:ODHCFS; Perceived social support:ad hoc tool; Cancer worry:CWS.	5
Van Oostrom et al., 2007b	NR	NR	Carrier-NonCarrier	T0: baseline – T1: 1 week – T2: 6 months after disclosure	Distress:IES; Anxiety and Depression:HADS; Nuclear family functioning:FACES; Differentiation to parents:DFSC; Familial communication style:ODHCFS; Perceived social support:ad hoc tool; Cancer worry:CWS.	5
Watts et al., 2011	Partner	Partner	NonCarrier	NR	Distress:DASS, IES; Dyadic adjustment:DAS; Support and team approach:ad hoc tool.	5
Wylie et al., 2003	Partner	Partner	Carrier-NonCarrier	T0: baseline – T1: 1 week – T2: 4 months – T3: 1 year – T4: 2 years after disclosure	Distress:IES; Spousal anxiety/distress; Family cancer history; Carrier status:ad hoc tool	5

Legend: NR = Not reported | AUS = Australia; CAN = Canada; JAP = Japan; NLD = The Nederland; UK = The United Kingdom; USA = The United states of America; PT = Portugal | GT = Genetic test; HNPCC = Hereditary nonpolyposis colorectal cancer; HBOC = Hereditary breast and ovarian cancer syndrome | FM = family member; MC =

pathogenic variant carrier | RMB = Risk management behaviours | BSI = Brief Symptom Inventory; CES-D = Center for Epidemiology Studies; CWS = Cancer Worry Scale; DAS = Dyadic Adjustment Scale; DASS = Depression, Anxiety, and Stress Scale; DCI = Dyadic Coping Inventory; : DCS = Decision Conflict Scale DFSS = Differentiation in the Family System Scale; DFSC = Decision Conflict Scale; DSI = Differentiation of Self Inventory; EPQ-R = Eysenck Personality Questionnaire; FACES = Family Adaptability and Cohesion Evaluation Scales; FES = Family Environment Scale; FRI = Family Relationship Index; HADS = Hospital Anxiety and Depression Scale; MSPSS = Multidimensional Scale of Perceived Social Support; HSCL = Hopkins Symptom Checklist; IES = Impact of Events Scale; IMI-C = Impact Message Inventory—Circumplex; LCWS = Lerman Cancer Worry Scale; MBSS = Miller Behavioural Style Scale; NEO-FFI = NEO-Five Factor Inventory; ODHCFS = Openness to Discuss; HCFS = Hereditary Cancer in the Family Scale; PAC = Parent-Adolescent Communication; PAS = Psychological Adaptation Scale; PRCI = Partner Response to Cancer Inventory; SAS-C = Support Actions Scale-Circumplex; STAI = Stait-Trait Personality Inventory; SCID = Structured Clinical Interview | 5 = 100% quality criteria met; 4 = 80% quality criteria met.
